# A Multifaceted Analysis of Immune-Endocrine-Metabolic Alterations in Patients with Pulmonary Tuberculosis

**DOI:** 10.1371/journal.pone.0026363

**Published:** 2011-10-13

**Authors:** Natalia Santucci, Luciano D'Attilio, Leandro Kovalevski, Verónica Bozza, Hugo Besedovsky, Adriana del Rey, María Luisa Bay, Oscar Bottasso

**Affiliations:** 1 Instituto de Inmunología, Facultad de Ciencias Médicas, Universidad Nacional de Rosario, Rosario, Argentina; 2 Instituto de Investigaciones Teóricas y Aplicadas, Escuela de Estadística, Facultad de Ciencias Económicas y Estadística, Universidad Nacional de Rosario, Rosario, Argentina; 3 Institute of Physiology and Pathophysiology, Marburg, Germany; National Council of Sciences (CONICET), Argentina

## Abstract

Our study investigated the circulating levels of factors involved in immune-inflammatory-endocrine-metabolic responses in patients with tuberculosis with the aim of uncovering a relation between certain immune and hormonal patterns, their clinical status and *in vitro* immune response. The concentration of leptin, adiponectin, IL-6, IL-1β, ghrelin, C-reactive protein (CRP), cortisol and dehydroepiandrosterone (DHEA), and the *in vitro* immune response (lymphoproliferation and IFN-γ production) was evaluated in 53 patients with active untreated tuberculosis, 27 household contacts and 25 healthy controls, without significant age- or sex-related differences. Patients had a lower body mass index (BMI), reduced levels of leptin and DHEA, and increased concentrations of CRP, IL-6, cortisol, IL-1β and nearly significant adiponectin values than household contacts and controls. Within tuberculosis patients the BMI and leptin levels were positively correlated and decreased with increasing disease severity, whereas higher concentrations of IL-6, CRP, IL-1β, cortisol, and ghrelin were seen in cases with moderate to severe tuberculosis. Household contacts had lower DHEA and higher IL-6 levels than controls. Group classification by means of discriminant analysis and the k-nearest neighbor method showed that tuberculosis patients were clearly different from the other groups, having higher levels of CRP and lower DHEA concentration and BMI. Furthermore, plasma leptin levels were positively associated with the basal *in vitro* IFN-γ production and the ConA-driven proliferation of cells from tuberculosis patients. Present alterations in the communication between the neuro-endocrine and immune systems in tuberculosis may contribute to disease worsening.

## Introduction

Tuberculosis (TB) is one of the most important infectious diseases and cause of death around the world. It is estimated that 2 billion persons are infected with *Mycobacterium tuberculosis*, and 8 to 12 million new cases of active tuberculosis occur each year, accounting for 2–3 million deaths annually [1].

Infection, which is acquired by inhaling air-borne droplet nuclei containing *M. tuberculosis*, usually takes place in the lungs, and begins as an alveolar non-specific inflammatory reaction that progresses to a typical delayed type granulomatous reaction. Most of the people infected with *M. tuberculosis* have a clinically latent infection, which remains dormant through mechanisms that prevent bacillary proliferation constituting asymptomatic and non-contagious latent carriers [2].

The development of clinical post-primary TB occurs in 5%–10% of latently infected persons, due to reasons that are not completely understood [3]–[5]. Factors able to affect host immunity such as malnutrition, alcoholism, advanced age, diabetes, and immunosuppressive drug treatments, among others, may be implied in this phenomenon [6]. Pulmonary disease is the most common form of post-primary TB. The response to bacillary multiplication is implied in the inadequate evolution of granulomas, provoking caseous necrosis followed by cavity formation through which infectious material can spread through bronchi and eliminated with the sputum [7]. Pulmonary TB presents a great spectrum of manifestations, ranging from few foci in the upper lobes or apical segments of inferior lobes to bilateral involvement with strong tissue damage. Patients often begin with an insidious clinical onset, progressing to typical manifestations as the disease progresses. Among them, cough, expectoration, lack of appetite, low-grade evening fever, night sweat and loss of body weight are frequently observed.

Tuberculosis offers an attractive model to investigate the pathological processes occurring during an infectious disease. First, the disease itself still poses a significant health and economic burden worldwide [1]. Second, the existence of metabolic-related manifestations [8], provides an extraordinary opportunity to study the immune-endocrine component that may underlie these alterations, considering that a reasonably stable energy supply is necessary to preserve all biological functions, for example the immune response [9], [10].

The metabolic status of an organism is finely regulated by the nutritional status, energy expenditure and hormonal signals. Our studies in TB patients revealed a series of immune and endocrine alterations characterized by increased levels of IFN-γ, IL-10, IL-6 and growth hormone, reduced amounts of testosterone and DHEA, in parallel to modest increases in the concentrations of cortisol, estradiol, prolactin, and thyroid hormones with no changes in insulin-like growth factor-1 [11]. We have further demonstrated that the weight loss in TB patients was related to the immune and endocrine disturbances, since the body mass index (BMI) was negatively associated with IL-6 circulating levels, whereas the levels of this cytokine correlated positively with cortisol concentrations [12].

Beyond these facts, it seems clear that these components may be only part of the disturbed metabolism during TB. The interaction between general metabolism and the immune response also comprises a series of pleiotropic products able to modulate appetite, energy expenditure, endocrine functions, inflammation and immunity, finally affecting the regulation of energy sources and immune activity [13]. Mediators of the inflammatory and specific immune responses (i.e., pro-inflammatory cytokines, acute-phase proteins) and factors involved in the neuro-endocrine regulation of food consumption like adipocytokines and the orexigenic hormone ghrelin should also be considered.

Herein we have explored several mediators participating in the immune-endocrine-metabolic unit and examined whether there is a relation between certain immune-endocrine patterns and the clinical status of TB patients, particularly weight loss. Healthy household contacts (HHC) of TB patients were also included as both groups constitute “natural models” for analyzing this type of immune-endocrine-metabolic relation. Assessment focused on circulating levels of leptin, adiponectin, IL-6, IL-1β, ghrelin, C-reactive protein (CRP), cortisol and DHEA.

Because of the need to understand the defensive response in an integrated fashion, an approach enabling to analyze several variables simultaneously was employed. The method is appropriated to find a combination of variables that allows to classify subjects into a correct group, or to identify the most suitable variables within the whole set of them.

Endogenous cytokines, hormones and adipocytokines are influential in the orchestration of the cellular immune response. Thus, the relation between plasma levels of such compounds and the *in vitro* immune response of TB patients was also analyzed.

## Materials and Methods

### Ethics statement

The study was conducted according to the Helsinki declaration and the protocol was approved by the Ethical Committee of the Facultad de Ciencias Médicas, Universidad Nacional de Rosario. Participants were enrolled, provided informed written consent had been obtained.

### Study Groups

Patients (20 females and 33 males) with no HIV co-infection and newly diagnosed pulmonary TB were included. Diagnosis was based on clinical and radiological data together with the identification of TB bacilli in sputum. Age of the patients ranged from 18 to 71 years -one case- (37±16 years, mean ± standard deviation). Disease severity was determined according to the radiological pattern and was classified into mild (n = 12) moderate (n = 19) or advanced (n = 22). A group of 27 HIV-1 seronegative HHCs was selected from first-grade contacts of acid-fast bacilli–positive TB patients. Each HHC shared the same house or room with an index patient for at least 3 months before the patient’s TB diagnosis. Household contacts were subjected to careful evaluation to discard tuberculosis, on the basis of clinical and radiological examinations (frontal and lateral chest X rays). The control population was composed of 25 healthy hospital controls, sharing the same socioeconomic conditions of HHC and TB patients, without any known prior contact with TB patients, as well as clinical or radiological evidence of pulmonary TB. HHCs and healthy controls revealed no statistical differences in age and sex distribution, and none had other respiratory disease, or immunocompromising diseases or therapies. BMI was calculated as weight/height^2^ (kg/m^2^). Nearly all subjects were BCG vaccinated with PPD positive reactions.

Blood samples were obtained from all donors at entry into the study; in TB patients before initiation of antituberculous treatment. Samples were obtained at 8 a.m. to avoid differences due to circadian variations. Exclusion criteria included disease states that affect the adrenal glands, the hypothalamus-pituitary-adrenal or hypothalamus-pituitary-gonadal axes, or requiring corticosteroid treatment, pregnancy, and age below 18 years.

### Mononuclear cell isolation, *in vitro* stimulation and cytokine measurements

Peripheral blood mononuclear cells (PBMC) were obtained from fresh EDTA-treated blood. After centrifugation the buffy coat was separated and diluted 1∶1 in RPMI 1640 (PAA Laboratories GmbH, Austria) containing standard concentrations of L-glutamin, penicillin, and streptomycin (culture medium, CM). The cell suspension was layered over a Ficoll-Triyosom gradient (density 1.077, Amersham Biosciences, NJ, USA) and centrifuged at 400 *g* for 30 min at room temperature (19–22°C). PBMC recovered from the interface were washed three times with CM and resuspended in CM containing 10% heat-inactivated pooled normal AB human sera (PAA Laboratories GmbH, Germany). Cells were cultured in quadruplicate in flat-bottomed microtiter plates (2×10^5^ cells/well in 200 µl) with or without addition of antigen obtained by sonication of heat-killed H37Rv *M. tuberculosis* (WSA; 8 µg/ml, kindly provided by Dr. J.L. Stanford, London) or concanavalin A (ConA; 2.5 mg/ml). PBMC cultures were incubated for 5 days at 37°C, in a 5% CO_2_ humidified atmosphere and pulsed with ^3^H-thymidine for 18 hr before cell harvesting.

To evaluate cytokine production, 10^6^ cells/ml were cultured with or without addition of WSA (8 µg/ml). Culture supernatants were collected after 24 and 96 hours and stored at −20°C until used for cytokine determinations. The concentration of IFN-γ in the supernatants was determined by ELISA using commercially available kits (OptEIA Set, Pharmingen, USA), and following the prescriptions provided by the manufacturer. Samples were assayed in duplicate and results are expressed as the average of the two determinations. Cytokines were quantified with reference to standard curves generated using human recombinant cytokines.

### Plasma collection and cytokine and hormone assays

Plasma was obtained from EDTA-treated blood. Samples were centrifuged at 2000 rpm during 30 min and the plasma stored at −20°C. Leptin (Quantikine, R&D Systems, detection limit 7.8 pg/ml, adiponectin (Quantikine, R&D systems, detection limit 3.9 ng/ml), Ghrelin (DRG Systems, detection limit 100 pg/ml), cortisol (DRG Systems, detection limit 20 ng/ml), DHEA (DRG Systems, detection limit 0.37 ng/ml), IL-1β (IL1-β Ultrasensible, Invitrogen, detection limit 0.2 pg/ml), and IL-6 (High Sensitivity (h)IL-6 Biotrack ELISA system, Amersham Biosciences, detection limit 0.63 pg/ml) plasma concentrations were determined using commercially available ELISA kits according to the manufacturer instructions. All samples were processed individually and assayed in duplicate. CRP plasma levels were determined by PCR Ultrasensible Turbitest, Wiener Lab, Argentina, detection limit: 3.3 µg/ml.

### Statistical Analysis

Since the distribution of variables was slightly skewed, statistical tests were non parametric. Summary statistics for all variables were calculated to compare groups. An initial univariate Kruskall Wallis analysis of variance was carried out to determine difference between group distributions. Post hoc paired comparisons with Bonferroni method were done for the variables that showed difference between groups. Spearman correlation coefficients between all variables were tested. To use data from all variables (age, leptin, adiponectin, CRP, DHEA, ghrelin, cortisol, IL-6, IL-1β and BMI) for characterizing each group and to find a function that enabled to classify individuals in one of the groups, a discriminant analysis was performed [14]. Prior probabilities proportional to the group sample sizes were used. Because of some limitations concerning the normal distribution of variables, outliers and different variance between groups, the nonparametric k-nearest neighbor method was also applied to classify individuals in each group [15]. Basically, the k-nearest method does not assume any special distribution of the variables and individuals are classified into the same class as its nearest (k) neighbors. A value of k = 3 and Mahalanobis distance to define nearness were used. Associations between hormone levels and the *in vitro* mycobacterial-driven immune response (lymphoproliferation and cytokine production) were analyzed by non parametric methods. Previous data by measuring cortisol and DHEA levels in TB patients revealed significant between-group differences in both cases, with a lower effect size in the case of cortisol. According to this, it was estimated that for a significance level of 5%, a sample size of 50 patients and 50 non diseased individuals had a little higher 95% power to detect differences. *P* values <0.05 were considered statistically significant. All statistical analysis was performed with SPSS v15.0 and SAS System v9.2.

## Results

### Cytokine and hormone levels in TB patients, HHC and healthy controls

We first analyzed the levels of several mediators known to participate in the immune, endocrine and metabolic responses. As shown in Table 1, it was clear that the BMI, leptin, CRP, DHEA, IL-6, cortisol, and IL-1β concentrations and the ratio cortisol/DHEA attained highly significant between-group differences, with adiponectin nearly reaching statistical significance. Comparisons of the leptin/BMI ratio, as an attempt to adjust for the effect of BMI influence on leptin levels, also revealed a significant difference. Post hoc paired comparisons with Bonferroni method for the variables showing between-group differences revealed that HHC had slightly higher levels of IL-6 than controls (Table 1). Most differences remained significant when comparing TB patients with either HHC or controls, except for the levels of DHEA levels in the two former groups. Overall comparisons among TB patients also revealed significant differences in the levels of leptin, ghrelin, IL-6, CRP, IL-1β, cortisol, as well as BMI, leptin/BMI ratio and Cortisol/DHEA ratio. When performing post hoc analysis, significant differences were found between mild vs. moderate cases (leptin, leptin/BMI ratio, CRP, ghrelin, IL-6, IL-1β), or mild vs. severe cases (BMI, leptin, leptin/BMI ratio, CRP, cortisol, cortisol/DHEA, IL-6 and IL-1β). In other words, BMI, leptin levels and leptin/BMI ratio decreased as disease severity increased, whereas higher concentrations of IL-6, CRP, IL-1β, cortisol, Cortisol/DHEA ratio and ghrelin were seen in cases with moderate to severe TB (Table 1).

**Table 1:** Levels of cytokines, adipocytokines, CRP, ghrelin and HPA related hormones in TB patients, household contacts (HHC) and healthy controls.

Variables	Patients					HHC(n = 27)	Controls(n = 25)	Overall*p* value
	Total(n = 53)	Mild(n = 12)	Moderate(n = 19)	Severe(n = 22)	*p* value(TB cases)			
Age	33(24–50.5)	28.5(21.5–44)	32(24–61)	35(27–54)	Ns	46(30–57)	34(24–45)	ns
(Females, %)	20 (38%)	5 (40%)	6 (32%)	9 (40%)	Ns	16 (59%)	13 (52%)	ns
BMI (kg/m^2^)	20.7(19.3–23.4)	22.2(20.3–24)	21.7(19.8–24.5)	19.6(18.5–21.4)‡	.015	27(23.6–30.4)	28.4(24.4–31.9	.0001*
Leptin (ng/ml)	1.60(0.76–3.32)	3.46 (1.97–16.41)	1.39(0.6–2.67)§	1.46(0.64–2.33)‡	.009	5.43(2.82–31.03)	7.01(4.71–16.53)	.0001*
Leptin/BMI ratio^a^	74.6(4.7–1022)	141.5(50.9–1022)	55.5(4.7–694.3)§	73.5(11.3–192)‡	.02	297(37.7–2336)	247(25–1895)	.0001*
Adiponectin (µg/ml)	10.26(7.07–23.14)	9.47(7.06–11.37)	12.02(7.91–18.6)	10.625(6.45–12.97)	ns	7.69(5.95–13.42)	6.70(4.74–9.515)	.056
CRP (mg/ml)	41.9(8.6–49.4)	8.4(3.5–13.7)	41.9(26.9–60.6)§	49.4(49–81.8)‡	.004	3.5(3.3–3.9)	3.3(3–3.5)	.0001*
DHEA (ng/ml)	3.6(2.2–5.3)	3.9(2.9–4.8)	4.8(2.2–6.2)	2.8(1.9–4.4)	ns	4(2.8–8.9)	6.6(4.5–10.1)	.001*
Ghrelin (ng/ml)	314.8(196–630)	199.8(178–347.5)	526.6(262–911)§	290(197–646)	.045	229(143–501)	251(198–479)	ns
Cortisol (ng/ml)	142.4(101–197.7)	106(52–159)	149(109–193)	151(115–287)‡	.04	113(66–139.8)	123(90.4–181)	.03*
Cortisol/DHEA	37.1(21.3–65.3)	22(17–346)	29(21–459)	54.6(38.3–107.9)‡	.0025	20.5(12.3–34.5)	17.7(13.4–23.1)	.0001*
IL-6 (pg/ml)	6.36(2.42–10.3)	2.1(1.81–3.14)	10.6(6.21–16.4)§	6.38(6.3–6.64)‡	.0007	1.12(1.09–2.17)¶	1(0.81-1-15)	.006*
IL-1β (pg/ml)	0.34(0.2–0.44)	0.2(0.2–0.24)	0.33(0.2–1.05)§	0.34(0.2–0.45)‡	.017	0.2(0.2–0.26)	0.2(0.2–0.26)	.001*

Values represent median (25–75 percentiles), data were analyzed by the Kruskall-Wallis non parametric analysis of variance.

a Calculated with leptin values in pg/ml.

* Differences remained significant when comparing TB patients either with HHC or controls, except for the levels of DHEA in the two former groups (p<0.05, post hoc Bonferroni comparisons).

¶ Significantly different from controls (*p*<0.05, post hoc Bonferroni comparison).

§ Significantly different from mild patients (*p*<0.05, post hoc Bonferroni comparisons within TB patients).

‡ Significantly different from mild patients (*p*<0.05, post hoc Bonferroni comparisons within TB patients).

Analysis of immune-endocrine data within HHC and control subjects classified according to a BMI below or above the median values revealed no differences (data not shown). Patients, HHCs and healthy controls presented no statistically significant differences as to age and sex.

### Correlation analyses between circulating levels of immune-endocrine mediators and BMI

According to the study purposes, we next performed correlations between circulating mediators and BMI. Data are referred to correlations that continued to be significant upon adjustment for multiple comparisons. This analysis is summarized in Table 2, which shows overall calculations and results obtained within TB patients and not ill individuals (controls and HHC). It can be seen that leptin positively correlated with BMI, also in TB patients and not ill individuals. By opposite, negative correlations between adiponectin, IL-6, IL-1β and CRP concentrations with BMI were found in the overall population. At a general level, leptin correlated negatively with IL-6 (r: −0.41, *p*<0.0001) and CRP (r: −0.39, *p*<0.0001).

**Table 2:** Correlation analysis of hormone and cytokine plasma levels with BMI in TB patients, healthy controls and household contacts (HHC).

Pair	Overall		TB patients		Controls + HHC	
	r	*p* value	r	*p* value	r	*p* value
Leptin vs. BMI	0.55	.00001	0.36	.01	0.43	.004
Adiponectin vs. BMI	-0.35	.0005				
IL-6 vs. BMI	-0.47	.00001				
IL-1β vs. BMI	-0.28	.007				
CRP vs. BMI	-0.48	.00001				

### Results from discriminant analysis

**Figure 1:**
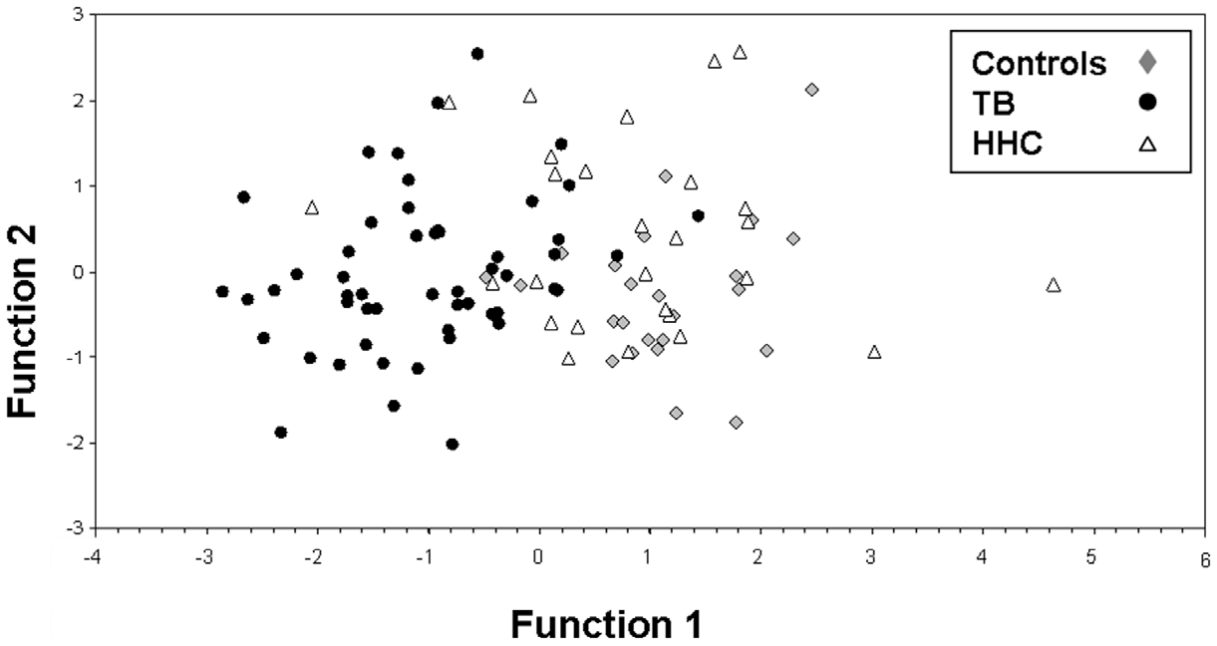
Plot of two linear discrimination functions of the model differentiating the study groups. Function 1 = 0.692*BMI - 0.655*CRP + 0.436*Leptin - 0.305* IL-6 - 0.185*Ghrelin - 0.124*IL-1β + 0.059*Age + 0.309*DHEA - 0.192*Adiponectin - 0.212*Cortisol. Function 2 = 0.036*BMI - 0.075*CRP + 0.368*Leptin + 0.117*IL-6 - 0.066*Grhelin -0.053*IL-1β + 0.691*Age - 0.562*DHEA + 0.406*Adiponectin - 0.305*Cortisol.

A discriminant analysis was performed to investigate which combination of immune-endocrine mediators could help to distinguish groups. The first linear discrimination function showed that the variables analyzed significantly differentiate groups, and the largest difference was observed between TB patients and non-TB subjects (Table 3 and Figure 1). According to the standardized coefficients, the most important variables in the first linear discrimination function (TB vs. the remaining groups), were CRP (−0.634), BMI (0.553), and DHEA (0.370), in which TB patients had high values of CRP, and low values of BMI and DHEA. The second linear discrimination (HHC vs. controls) indicated a trend statistically non significant wherein HHC presented higher values of leptin and adiponectin and lower values of DHEA and BMI than controls. As depicted in Table 3, correct classification of the model is 74.3% (62.9% cross-classification). Visual inspection of the discriminant functions’ territory map revealed that the model can clearly differentiate TB patients from the two other groups but classification between HHC and controls is not as clear (Figure 1).

**Table 3:** Results from discriminant analysis.

Procedure	Group	Predicted Group Membership			Total
		Controls	HHC	TB	
Original	Controls	16 (64)	6 (24)	3 (12)	25
	HHC	7 (26)	13 (48)	7 (26)	27
	TB	0 (0)	4 (7.5)	49 (92.5)	53
Cross-validated a	Controls	12 (48)	10 (40)	3 (12)	25
	HHC	8 (29.7)	9 (33.3)	10 (37)	27
	TB	1 (1.9)	7 (13.2)	45 (84.9)	53

Numbers between parentheses represent percent values respect the total number per group.

a Leave-one-out cross validation is done in the analysis: each observation is classified by functions derived from all observations other than that observation.

74.3% of observations correctly classified.

62.9% of observations correctly classified with cross-validation.

Discriminant analysis works best for normal distribution and homoscedasticity between groups. Since our data do not completely fulfill these characteristics and some outliers were present, an alternative non-parametric method was applied to improve the correct classification. As can be seen in Table 4, correct classification by the k-nearest neighbor (with k = 3) achieved 78.3%; with 52%, 91% and 80% of HHC, TB patients and controls, being correctly classified, respectively. Again, it can be also appreciated that the method clearly differentiates TB patients from the other two groups, but classification for HHC and controls is not as clear. Cross-classification analysis revealed a 69% correct classification (Table 4 second part).

**Table 4:** Classification by the non parametric method of nearest neighbors (k = 3, taking the three nearest neighbors).

Procedure	Group	Predicted Group Membership				Total
		Controls	HHC	TB	Undefined	
Original	Controls	20 (80)	1 (4)	1 (4)	3 (12)	25
	HHC	6 (22.2)	14 (51.9)	4 (14.8)	3 (11.1)	27
	TB	0 (0)	2 (3.8)	48 (90.5)	3 (5.7)	53
Cross-validated	Controls	15 (60)	7 (28)	3 (12)	-	25
	HHC	10 (37)	9 (33)	8 (30)	-	27
	TB	0 (0)	5 (9)	48 (91)	-	53

Numbers between parentheses represent percent values respect the total number per group.

78.3% of original grouped cases correctly classified.

69% of cross-validated grouped cases correctly classified.

### Relation between circulating levels of immune-endocrine mediators and the mycobacterial-driven immune response

**Figure 2:**
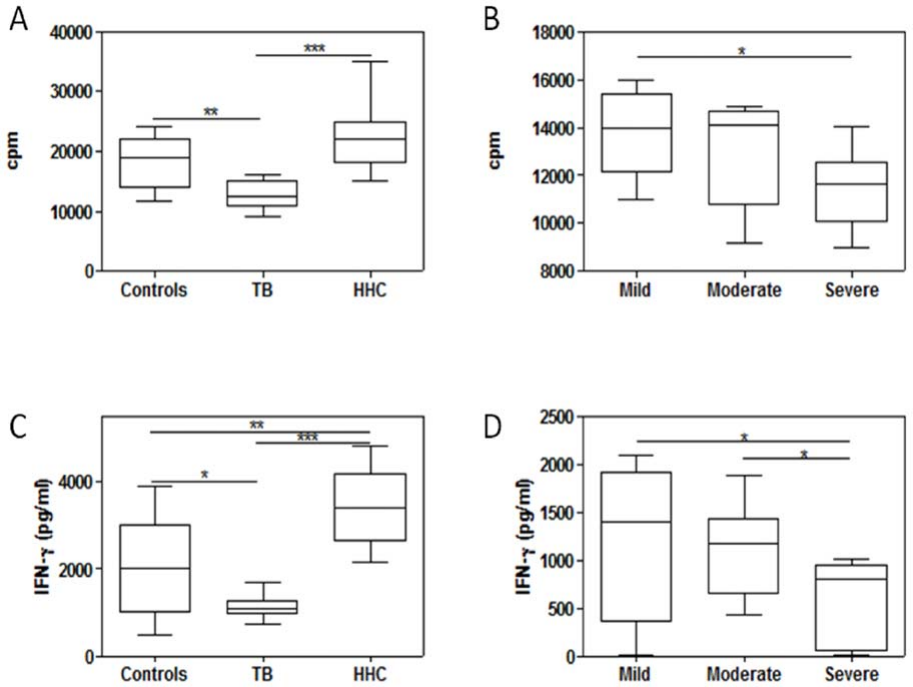
Proliferative responses to mycobacterial stimulation and levels of IFN-γ in 96-h culture supernatants of peripheral blood mononuclear cells from TB patients (n = 47), household contacts (HHC, n = 22) and healthy controls (n = 23). Patients were separated according to disease severity into mild (n = 10), moderate (n = 18) and severe (n = 19) cases. Box plots show 25–75 percentiles of data values in each group with maximum and minimum values. The line represents the median values. Panels A and C: lymphoproliferative response and IFN-γ production in TB patients, HHC and controls, respectively; Panels B and D: lymphoproliferative response and IFN-γ production in TB patients according to severity, respectively. Overall comparisons by the Kruskall-Wallis test revealed significant differences in lymphoproliferation and 4-day IFN-γ production (*p*<0.05 and *p*<0.02, respectively). Significant post hoc Bonferroni comparisons between groups and within TB patients are depicted by *, ** and ***, *p*<0.05, p<0.025 and *p*<0.01, respectively.

In line with our former observations on the *in vitro* mycobacterial-driven immune responses [16], HHC and controls had easily noticeable *in vitro* responses to mycobacterial stimulation, whereas TB patients had an overall diminished lymphoproliferation and reduced 4-day IFN-γ, yielding statistically significant differences in both overall comparisons (Figure 2, panels A and C). Post hoc paired comparisons with the Bonferroni method revealed that HHC and controls had significantly higher lymphoproliferation and IFN-γ production than TB patients, with HHC also showing a significantly increased synthesis of this cytokine in relation to controls (Figure 2, panels A and C). Overall comparisons among TB patients showed significant differences in mycobacterial-induced lymphoproliferation and IFN-γ production (Figure 2, panels B and D). This was at the expense of even lower responses in severe cases, significantly different than those seen in mild (lymphoproliferation and IFN-γ production) and moderate cases (IFN-γ production) cases when performing post hoc comparisons.

Mitogen-driven proliferation was also significantly diminished in TB patients, and comparisons among the group of TB patients showed lower lymphoproliferation and IFN-γ production in severe cases (data not shown).

Analysis of the relation between circulating levels of immune and endocrine mediators and lymphoproliferation and cytokine production was carried out by calculating pair wise correlations. Results revealed that plasma leptin levels were positively correlated with the basal amount of IFN-γ present in 24 h culture supernatants from TB patients (r: 0.68, *p*<0.029). A positive correlation between leptin levels and ConA-driven proliferation was also found (r: 0.65, *p*<0.05).

There were no significant correlations when the levels of other mediators (adiponectin, IL-6, IL-1β, CRP, and ghrelin) were compared with *in vitro* cytokine production or lymphoproliferation.

## Discussion

The control of an infectious process depends on the quality and magnitude of the defensive response. This is primarily mediated by immune-associated mechanisms, in combination with additional factors that favor a fine tuning of protective mechanisms by influencing the microenvironment in which the immune cells exert their functions. When the pathogen cannot be properly eradicated, the same mechanisms employed for protection turn out to be detrimental for the host and hence implied in disease pathology. The results of the present study indicate that pulmonary TB coexists with important immune, endocrine, and metabolic changes; some of them clearly associated with the BMI, which in turn is a main manifestation that reflects the clinical status of patients. To our knowledge, this is the first report performing a multifaceted analysis of classical and novel mediators involved in such relation during protective and disease states of *M. tuberculosis* infection.

Overall energy homeostasis depends on the nutritional status, energy expenditure and hormonal signals. Several compounds, such as adipocytokines and the orexigenic hormone ghrelin, participate in the neuro-endocrine regulation of these responses. Leptin is recognized as a hormone produced by white adipose tissue and involved in the control of energy storage by decreasing food intake and increasing energy expenditure [17], [18]. Determinations of leptin in plasma of TB patients have yielded dissimilar results. While some reports showed increased leptin levels in TB patients [19], other studies reported no changes [20] or decreased concentrations associated with decreased body fat and loss of appetite [21]. Schwenk et al. [22] suggest that leptin may not be involved in the loss of weight in TB, although in their study leptin positively correlated with body mass. To some extent, considering the already known correlation between serum leptin levels and BMI [23], and that leptin concentration is related to the adipose tissue mass [24], the reduced leptin levels found in this and in a former series of TB patients [25] may be linked to the weight loss that accompanies the disease. At the same time, it is known chronic stimulation with pro-inflammatory cytokines, as is the case of TB, suppresses leptin production [26], [27]. The fact that HHC, who undergo a latent subclinical tuberculous infection, showed no weight loss and shared the same socio-economic and environmental conditions, had normal leptin plasma levels, favors the view that reduced leptin levels in TB are more likely due to an energy imbalance. Reinforcing this view, the leptin/BMI ratio was equally reduced in TB patients. The typical loss of appetite of TB patients in the presence of decreased amounts of leptin also suggests that this counter-regulatory mechanism for food intake is inefficient here and that other factors are involved in this phenomenon.

Adiponectin and ghrelin are known to participate in the regulation of food consumption. Adiponectin is related to increase in appetite, central fat distribution and multiple metabolic disorders [28]. Ghrelin, the endogenous ligand of the growth hormone secretagogue receptor, is a potent circulating orexigen, and controls energy expenditure [29], [30]. This complementary activity of leptin, ghrelin and adiponectin in providing information to the CNS about the energy balance to maintain homeostasis, seems to be disrupted in TB since their circulating pattern is compatible with an orexigenic effect.

Very recently, Kim at al. [20] reported no differences in leptin and ghrelin levels in TB patients, although further separation into well-nourished or malnourished cases revealed lower amounts of ghrelin in the latter subgroup. Reasons for differences between this and our study findings may be of different nature. Our sample population was composed of untreated patients whereas in the Korean study patients were bled within 3 days following treatment initiation. Additional factors, i.e, genetic and environmental or predisposing factors may be also implied. In distinguishing prior malnutrition from the consumption state that accompanies progressive disease, the preserved nutritional status of close HHC of present TB patients adds support to the latter possibility.

Whatever the case, it may be assumed that anorexia and the consumption state during TB are the result of a complex network of mediators with additive, interactive, and antagonistic interactions, in a not necessarily unique scenario. A candidate to play a role in these interactions is IL-6. As seen in a former study [12], IL-6 levels are increased in TB patients and this cytokine is known to reduce retroperitoneal fat and circulating levels of leptin [31]. Our results are also in line with the findings from Stenlof et al. [32] reporting a negative correlation between IL-6 levels in cerebral-spinal fluid, body weight and serum leptin concentrations in obese patients. Another factor likely involved in the catabolic state may be IL-1β. This cytokine was not only increased during TB but also negatively correlated with the BMI. At the experimental level IL-1β is known to produce a profound hypoglycemia in mice and a re-setting of glucose homeostasis that favors fuel re-distribution towards immune cells but with a paradoxical reduction of food intake [33]. Moreover leptin actions on food intake and body temperature appear to be mediated by IL-1 [34].

In parallel, cortisol may favor the loss of body mass seen in the patients, since it mobilizes lipid stores by inducing lipolysis in fat cells inhibiting protein synthesis and stimulating proteolysis in muscle cells [35]. Increased cortisol levels may also be involved in the catabolic status by inhibiting food intake and thus inducing body weight loss [36]. In parallel to these alterations, TB patients displayed increased amounts of CRP, which were even higher in severe cases. This finding is in line with other studies reporting increased levels of CRP in HIV seropositive and seronegative patients with TB [37]–[40], and highlights the important degree of nonspecific systemic inflammation implied in this disease.

Correlation studies also allowed to identify two distinct types of associations between immune, endocrine and metabolic variables. On one hand is the positive correlation between BMI and leptin, and the negative relation of this adipocytokine with IL-6 and CRP. Conversely, adiponectin, IL-6, CRP and IL-1β were inversely correlated with the BMI.

In general terms, the present results differ from the reported effects of leptin, which is known to increase the production of several pro-inflammatory cytokines by monocytes/macrophages [41] as well as displaying some pro-inflammatory effects [26], [27]. At variance with a recent study demonstrating an inverse relation between plasma adiponectin levels and a number of inflammatory markers [42], in our case changes in adiponectin, IL-6 and IL-1β were in the same direction.

To some extent, this skewed profile of associations may be related to a dysregulation of a defensive response of TB patients, more evident in the case of leptin, with repercussions at the level of catabolism and protective immunity. In fact, leptin plasma levels positively correlated with *ex vivo* basal production of IFN-γ and ConA-driven proliferation by cells from TB patients. In addition to its metabolic effects, leptin is able to favor Th1 responses [26], [27]. Low endogenous levels of leptin in TB patients may partly account for the impaired specific cellular immune responses typically observed in these patients. The present results complement our previous demonstration that changes in the levels of adrenal steroids in plasma are paralleled by alterations in the mycobacterial-driven *in vitro* response of TB patients [43].

The aim of this work was to simultaneously analyze variables involved in the immune-endocrine-metabolic relation during TB by using two well-recognized methods, discriminant analysis and the nearest neighbor method. Both procedures allowed to differentiate well TB patients from not ill individuals, whereas discriminant analysis identified DHEA, CRP and BMI as the more significant variables to predict group designation within the whole set of estimations. The implications of weight loss and the BMI in TB were recently discussed (reviewed in [8] and [44]). DHEA is known to stimulate helper T-cell functions, facilitates Th1 responses and also exerts potent anti-inflammatory effects [45]. Our results are in line with data from patients with chronic disabling inflammatory diseases in whom a prolonged immune aggression coexist with a deficient production of adrenal steroids [46]. In other infectious diseases of chronic nature, i.e., leishmaniasis and Chagas disease, levels of DHEA were also found reduced [47], [48].

Several pieces of evidence from our group suggest that decreased DHEA levels in TB patients are unfavorable for the course of the disease, in terms of the disturbed anti-infective immune response, control of tissue damage, and metabolism [8], [44]. Supporting this view, treatment with a synthetic DHEA derivative improved the course of experimental tuberculosis in mice [49]. Also, administration of DHEA sulphate improved IgG and IFN-γ production in mice immunized with heat shock proteins from *M. tuberculosis* [50].

Discrimination methods were less effective in distinguishing HHC from healthy controls, for which the evaluation of additional variables may be required to achieve a clear-cut separation. Insulin and glucagon levels remained within the normal range in all groups (data not shown). Nevertheless, a trend of HHC to display increased adipocytokines levels, together with lower amounts of DHEA and higher IL-6 concentrations (this mediator statistically significantly different from controls) was found. While meticulous clinical and radiological evaluation gave no indication of an overt disease, close contacts of TB patients are likely to course a subclinical TB infection [16], [51], and increased levels of IL-6 may reflect a subclinical inflammation.

An efficient communication between the neuro-endocrine and immune systems is essential to maintain homeostasis and health [52]. In cases in which the noxious stimulus cannot be eliminated, as in TB, our study suggests that the resultant of the host response to such aggression increases the risk of worsening the disease. The results also indicate that a multi-mediator approach is suitable for a more integrated view of the pathogenesis of the disease.

Findings are relevant as they come from what is occurring in real situations.
